# The Cause of Milk Sickness

**Published:** 1888-02

**Authors:** C. D. Smith

**Affiliations:** Franklin, N. C.


### THE CAUSE OF MILK SICKNESS.

We take pleasure in publishing, and especially calling the attention of our readers to the following private letter addressed to one of the editors of this journal by Mr. C. D. Smith, of Franklin, North Carolina.

Mr. Smith is not a physician, but is a geologist of great distinction and of great practical experience in the line of his profession. He is especially familiar with the topography, mineralogy and flora of the entire mountain region from Virginia to the Alabama line. It is in this region that "milk sickness" is most frequently observed, and still causes, annually, many deaths.

We are not in a position to express an opinion in regard to the theory of the causation of this disease which Mr. Smith presents. It is, however, the conclusion, based on much observation by an intelligent and scientific layman, and deserves full investigation of medical men living in the section of country above indicated :

DEAR SIR—In a conversation sometime ago with Dr. A. C. Brabson, I learned from him that you were preparing a paper on the disease known as milk sickness. Some years ago I had a long interview with Dr. B., in which I communicated some information which I had collected, and the conclusions at which I had arrived. My theory was the result of an interview with Dr. H. G. Woodfin about milk sickness, during which he remarked that he could, in a few minutes after entering the room of a milk-sick patient, determine it by the peculiar odor which always attended that disease. I had just been reading up on the vegetable alkaloids, and had noted the peculiar odors which belonged to some of them. The more I thought about it the more I was impressed that the milk-sick poison might be a vegetable alkaloid. This was over thirty years ago. Adopting the theory that the disease was most probably due to a vegetable alkaloid, I set about collecting what information I could get from those who lived in milk-sick districts, and I pursued the investigation from Virginia to Georgia. From the general testimony elicited, the first conclusion at which I arrived is that the poison is a very volatile one, which is taken up by the milk absorbents during the process of *digestion.* In proof of this, those having experience with the disease assert positively that a cow, giving milk and having a sucking calf at the time of contracting the disease, will communicate it to the calf through the milk. When the calf dies, as it is most likely to do if the cow be frequently and thoroughly milked, the poison will, through the milk, be sufficiently drawn off to prevent the death of the cow, and she will recover. From this fact my conclusion was, and I still adhere to it, that the poison is a vegetable alkaloid, and the most reasonable theory which has presented itself to my mind is that it belongs to the family of the cryptogams. I suppose no one has ever attempted to enumerate the countless variety of the cryptogamous plants. Some are microscopic and the creature of a night, disappearing under the rays of the morning sun. Such variety, or a class of such variety, is most probably the source of the poison under consideration.

It is a well-established fact that "milk-sick" is contracted in deep, densely shaded north coves or other densely shaded localities where the accumulating vegetable matter is rarely burned off and is consequently left to decay upon the surface. Another pertinent fact is that the disease is never contracted only during the season of a warm, humid atmosphere. This atmospheric condition is particularly favorable to the production of some of the microscopic cryptogams, especially in the shaded localities referred to. The probability is that these cryptogamous plants possess a brackish taste which causes cattle wanting salt to lick it. In confirmation of this, those living in milk-sick regions assert that cattle which are kept abundantly salted are less subject to the disease. Then again I gathered this item of information from a family living in the mountains of Virginia. I was stopping with them and making an examination of some mineral lands. I observed that the milk-cows were in the pen at about 10 o'clock. I expressed my surprise, urging the point that cattle loved early grazing. The explanation was this: "There is milk-sick just back there in the cove, and we have learned that if we keep up the cows until the sunshine dries out the cove, there is no danger of their taking it." This testimony harmonizes with the suggestions already made. There is another fact which points to the same conclusion. After the warm season has passed and the season of white frosts comes in the disease is not contracted ; all danger from it is passed. It may possibly have been contracted in mild form during its proper season and not developed until after frost.

There is nothing, so far as I can see, in the information I gathered and the facts I have stated, that indicates the milk-sick poison to be a mineral or acid poison. It is most assuredly an alkaline poison, but I will not presume to speak of the practice of those physicians who understand the disease and who have uniformly treated it with marked success. Both Drs. Rush and Brabson understand the disease and the remedies thoroughly and can give you their treatment. I know nothing about the practice of medicine. My study of the subject was only incidental to my geological and mineralogical work in the field where the causes of the disease were known to exist.

Yours truly, C. D. SMITH.

*Franklin, N. C., December 28, 1887.*

